# A Critical Evaluation of Privacy and Security Threats in Federated Learning

**DOI:** 10.3390/s20247182

**Published:** 2020-12-15

**Authors:** Muhammad Asad, Ahmed Moustafa, Chao Yu

**Affiliations:** 1 Department of Computer Science, Nagoya Institute of Technology, Nagoya 466-8555, Japan; ahmed@nitech.ac.jp; 2 Faculty of Informatics, Zagazig University, Zagazig 44519, Egypt; 3 School of Data and Computer Science, Sun Yat-Sen University, Guangzhou 510275, China; yuchao3@mail.sysu.edu.cn

**Keywords:** federated learning, privacy, security, threats, attacks

## Abstract

With the advent of smart devices, smartphones, and smart everything, the Internet of Things (IoT) has emerged with an incredible impact on the industries and human life. The IoT consists of millions of clients that exchange massive amounts of critical data, which results in high privacy risks when processed by a centralized cloud server. Motivated by this privacy concern, a new machine learning paradigm has emerged, namely Federated Learning (FL). Specifically, FL allows for each client to train a learning model locally and performs global model aggregation at the centralized cloud server in order to avoid the direct data leakage from clients. However, despite this efficient distributed training technique, an individual’s private information can still be compromised. To this end, in this paper, we investigate the privacy and security threats that can harm the whole execution process of FL. Additionally, we provide practical solutions to overcome those attacks and protect the individual’s privacy. We also present experimental results in order to highlight the discussed issues and possible solutions. We expect that this work will open exciting perspectives for future research in FL.

## 1. Introduction

**Figure 1:**
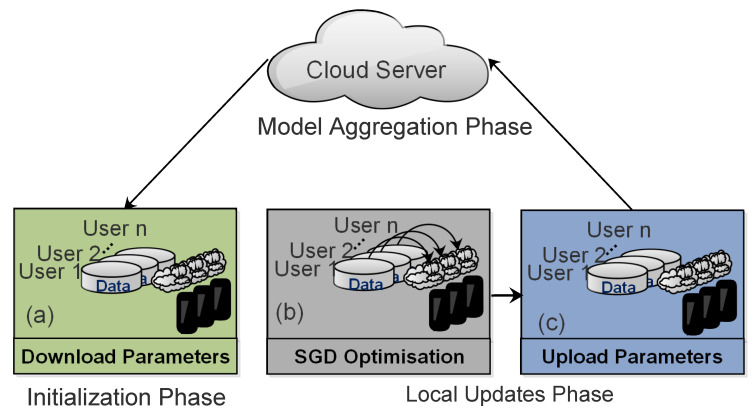
General framework of federated learning.

The Internet of Things (IoT) has achieved great popularity and acceptance with the rapid growth of high-speed networks and smart devices [1]. In this respect, IoT represents a network, in which “things” or devices are interconnected through a public or a private network. These devices are equipped with tiny sensors and powerful hardware that collect and process data at unprecedented speed [2]. On the other hand, Artificial Intelligence (AI) have simultaneously revolutionized the data extraction techniques with ground-breaking success in various applications, such as natural language processing [3], facial recognition [4], autonomous driving [5], and voice recognition [6]. Generally, those applications require clients to extract data from a distributed environment, which results in data privacy issues that have been a growing concern, recently [7]. In particular, centralized data repositories face major private data-leakage issues, e.g., travel information, health condition, and financial data [8]. Additionally, social networks and other data applications brought major privacy concerns for clients. These privacy concerns of clients are supposed to minimize by introducing a new paradigm, i.e., Federated Learning (FL) [9]. Specifically, FL allows for each client to collaboratively train an ML model on their private data without revealing the sensitive information to the centralized cloud server [10]. This collaborative training of the global model prevents direct data leakage from clients, which is a distinctive privacy advantage over the centralized training of data [11]. Figure 1 shows the general framework of FL. However, despite this secure training technique, the server could still put the client’s privacy at risk during the connection with other datasets. To this end, several approaches, including obfuscation methods, like differential privacy [12], secure multi-party computation (SMC) protocols [13], and cryptographic techniques, like homomorphic encryption [14], have been proposed. However, even with these efficient approaches, the adversaries can still reconstruct the raw data due to the shared architecture and not fully protected learning parameters. In addition, most of the FL approaches use optimization algorithms for updates, namely, stochastic gradient descent (SGD), which can leak the private information because of the direct transmission of gradient updates [15]. Furthermore, malicious users in the network may induce additional privacy poisons. Therefore, the design of secure FL architecture still needs further investigation on shared parameters for privacy and security protection. This paper focuses on this research challenge and investigates the potential privacy and security issues in FL.

The remainder of this paper is organized, as follows: Section 2 defines the overview of FL. In Section 3, we define the essential terminologies used in this paper. In Section 4, we summarize the existing literature, developments, and directions. In Section 5, we present our major contribution through experiments and show the impact of poisoning and inference attacks on each of the FL execution phases. In addition, we provide possible solutions to overcome the discussed attacks. In Section 6, we discuss few potential future directions of FL, which are yet to be properly discovered. Finally, in Section 7, we conclude this paper.

## 2. Overview of Federated Learning

The general framework of FL consists of multiple clients and a cloud server, where each client downloads a shared global model from the cloud server for the local training of data. Afterward, all of the clients periodically forward their locally trained models to the cloud server. The cloud server performs a global average and aggregates the improved global model to the clients. This communication between the clients and the cloud server (usually known as communication round) is continuously repeated until the desired convergence level is achieved [16]. The data distribution among clients in FL further classifies it into three categories; Horizontal Federated Learning (HFL), Vertical Federated Learning (VFL), and Federated Transfer Learning (FTL) [17]. Below, we define these three categories from security perspectives:

- Horizontal Federated Learning (HFL): the HFL is also known as sample-based FL. It is usually applied to the scenarios, where the datasets share the same feature space, but different space in samples. In HFL systems, an attack is usually assumed from the untrusted and curious cloud server, whereas the clients in HFL are considered honest.
- Vertical Federated Learning (VFL): the VFL is also known as feature-based FL. It is usually applied to the scenario where two datasets need to share identical sample IDs, but different feature spaces. In VFL systems, it is assumed that an adversary between two non-colluding parties can compromise a client’s privacy. The adversary learns the data from the corrupted party, while the data of the other party remains secure.
- Federated Transfer Learning (FTL): the FTL is usually applied to the scenario where two datasets are different in feature space and samples. The security concerns in FTL systems are the same as in VFL systems, because it also involves two non-colluding parties.

**Figure 2:**
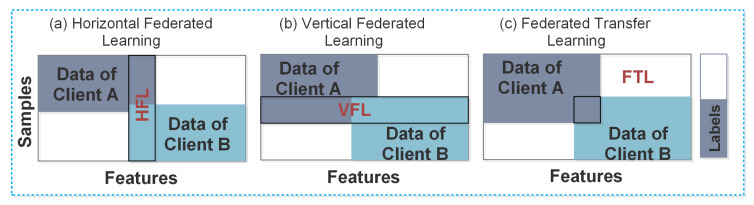
Classification of federated learning.

In Figure 2, we present the general classifications of FL. However, the design of this classification contains vulnerabilities for both the clients and cloud server. For example, the clients can observe the global parameters to control the uploads, i.e., malicious clients alter the inputs and infer the attacks through stealthy backdoors in the global model. Similarly, the cloud server can observe individuals’ updates, modify the training process and control the clients’ views on global parameters. These attacks from either side hinder FL to be implemented widely. Therefore, it is essential to understand the principles behind these attacks before implementing the FL protocols. This paper summarizes some of the critical threats in FL and provides possible solutions to prevent them.

## 3. Preliminaries

In this section, we define the essential terminologies in order to better understand the main contribution of this paper.

### 3.1. Privacy and Security

In the literature, privacy and security are used interchangeably; therefore, it is vital to know the difference between them. On the one hand, privacy issues refer to the unintentional disclosure of personal information, such as a harmless open dataset containing the individual’s personal information. Generally, privacy attacks only require common sense and they do not involve any hacking activities. In order to prevent privacy issues, we can use anonymity, un-observability, and un-linkability. On the other hand, security issues refer to the unauthorized/malicious activities, modification, or denial of data. Generally, security attacks are launched by trained hackers who have expert knowledge about the targeted system. To prevent security issues, we can use integrity, availability, and confidentiality [18].

### 3.2. Poisoning Attacks and Inference Attacks

In the general settings of FL, both the participants and cloud server are considered as honest-but-curious; honest because they do not deviate from the FL protocol and curious because they try to learn the private states of other parties. The curious participants or the curious cloud server may launch poisoning attacks or inference attacks in order to gain access to an individual’s private information. In general, the poisoning attacks refer to the modification of training data, which results in an alteration of the model’s behavior. These poisoning attacks can be random or targeted, which reduces the model’s accuracy or to induce the targeted label for the desirable output, respectively. In contrast, inference attacks refer to the leakage of private information during the exchange of gradient updates. In particular, adversaries can gain clients’ unintended features during the model updates, such as membership, class representatives, and the properties that are associated with the subset of training data. An adversary can also use the shared gradients and modify the labels in order to recover the original training samples.

## 4. Summary of Existing Studies

In the general settings of FL, clients’ private data are not exposed to adversaries without any external attack. However, the adversaries can launch malicious attacks to infer private information due to the curious nature. In the literature, many discovered attacks can potentially leak private data by accessing the FL model. The researchers also developed privacy mechanisms, such as differential privacy, cryptography, and hashing techniques, in order to guarantee the privacy of an individual’s information to prevent such attacks. In addition, the byzantine attack is also being considered in FL, where the malicious adversaries may behave arbitrarily and produce their outputs similarly to the correct model updates, which makes the aggregator hard to detect. Currently, there are two approaches that have been widely adopted in FL for privacy and security threats: differential privacy and cryptography techniques. Below, we define the methodology of these approaches:

- **Differential Privacy (DP):** the differential privacy (DP) adds the random calibrated noises to the data or the model parameters to guarantee that the output function does not influence a single record. In Table 1, several existing studies adopt DP for privacy protection, where the adversaries cannot get the knowledge, whether the record has participated in the learning. The random calibrated noises provide statistical privacy guarantees to the individual’s record and prevents the model from inference attacks. However, the noises in the learning process tend to produce less accurate models, because the accuracy is inversely proportional to the added noises.
- **Cryptography Techniques (CT):** the cryptography techniques, such as homomorphic encryption and secure multi-party computation (SMC), are widely used in the existing literature of privacy-preserving FL algorithms. In particular, each client encrypts the update before uploading it to the cloud server, where the cloud server decrypts these updates in order to obtain a new global model. However, these techniques are vulnerable to inference attacks, because each client has to share the gradients accessible to the adversaries. Applying cryptography techniques to the FL systems can also result in major computation overhead, due to the extra operations of encryption and decryption.

In addition to the above approaches, FL can adopt other techniques in order to guarantee privacy, such as hashing and incentive mechanisms. In Table 1, we summarize the state-of-the-art research contributions on privacy and security aspects, where most of the existing literature is focused on horizontal data partitioning (HFL). The major reason behind this is that the benchmarking and experimental studies in HFL are relatively ready as compared to vertical data partitioning (VFL). Therefore, VFL needs special consideration in the future, as it is also common in the real-world, especially between different organizations. Additionally, FTL has not been discovered for privacy-preserving techniques. We have analyzed that most of the research contributions have focused on two metrics privacy and efficiency in FL. Because FL is related to a large scope of applications, we believe that we will see more exciting and interesting studies in the future.

## 5. Critical Evaluation of FL

In this section, we conduct a critical evaluation of privacy and security threats in order to prevent the clients’ data from severe attacks, i.e., inference attacks and poisoning attacks during the execution of FL protocol in each phase. Firstly, we summarize the properties of these attacks in Table 2. Afterward, we explain the experimental setup. In the end, we demonstrate those threats through experimental results and provide a possible solution to avoid them in the following subsections.

### 5.1. Experimental Setup

In order to provide proof of threats, we conduct the experimental evaluation to demonstrate the privacy and security challenges in FL. The detailed experimental setup is explained experiment-wise in the following sub-sections; here, we define each experiment’s overall setup. We locally compute the SGD updates by partitioning the training dataset into disjoint non-IID training sets, and then we aggregate the updates while using the averaging method to train a globally shared classifier. In particular, we execute FL with separate non-IID training data. For example, we have *M* classes for the MNIST dataset; we form *G* group of clients’ devices and then evenly split the dataset on those clients’ devices. For the non-IID FL model, we assign a training instance with a label *m* to the *g*-th group with the probability *P*, where P>0. We call this probability a degree of non-IID, where a higher value of *P* indicates a higher degree of non-IID. All of the experiments are conducted on the server with an Intel(R) Core (TM) i5-9600K CPU @ 3.70 GHz, while TensorFlow implements the FL algorithm in Python. We repeat each experiment for at least five times and report the average result due to machine learning models’ randomized nature. Table 3 provides the hyper-parameters for all of the experiments.

**Dataset:** we use a publicly-available and commonly used dataset for FL: MNIST, a digit classification problem divided into 60,000 training samples and 10,000 testing samples. Each sample is based on images of handwritten digits ranging from 0–9 with a size of 28×28 pixels. The MNIST dataset is trained on the Convolution Neural Network (CNN) model with two 5×5 convolution layers, an output layer, and a fully connected layer. Our primary concern is to show how poisoning attacks or inference attacks disturb the convergence accuracy; therefore, the Deep Neural Network (DNN) architecture does not necessarily target the minimum loss in the considered dataset. Table 4 shows the complete architecture of the DNN model for the MNIST dataset. This DNN architecture does not necessarily achieve the lowest error for the MNIST dataset because the major goal of the experiments is to show the learned DNN classifier’s convergence behavior after launching the poisoning or inference attacks by the adversaries.

### 5.2. Initialization Phase: A Privacy Threat

At the beginning of the FL protocol, an untrusted centralized cloud server finalizes the training tasks, i.e., corresponding data requirements and the targeted application. The cloud server also specifies the training process, e.g., the learning rate and the global model’s hyper-parameters. After deciding all of these requirements for the FL task, the cloud server sends this information along with the initialized global model to all the clients in order to select the participants for the training. After receiving the acknowledgment from the clients, the cloud server then selects a certain number of participants. The selected clients can have the control to observe the model’s states, which allows for them to contribute arbitrary updates during the distributed training process. At this point, curious clients may have a chance to manipulate the training process with malicious activities. In particular, the malicious client can execute the poisoning attacks in order to modify the training datasets or alter the learning process’s integrity. Malicious clients can also launch an inference attack at the initialization phase in order to manipulate the individual’s update. For example, a client modifies a single label from samples of a specific class before learning, which results in poor performance of the model on this specific class.

#### 5.2.1. Experiment

**Figure 3:**
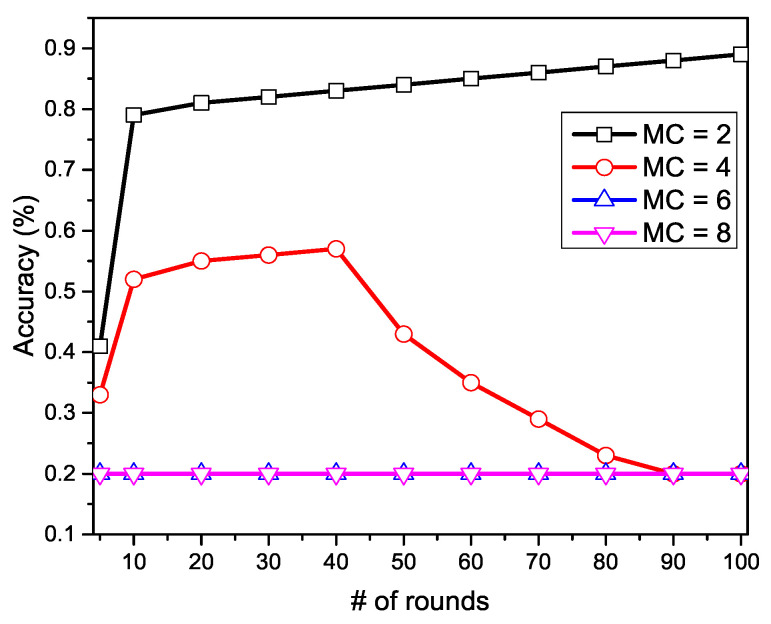
Privacy threat with various number of malicious clients at initialization phase.

In the initialization phase, the private information of clients can only be compromised by malicious participants. Therefore, in Figure 3, we show the performance comparison with four different numbers of malicious clients: MC={2,4,6,8}. In particular, we deploy 20 clients, in which the malicious clients tend to upload the fake value of model parameters in each communication round, where the fake value is the opposite of the true value or a random number ranging between −1 to 1. Figure 3 demonstrates that malicious clients’ involvement badly influenced the performance and it kept decreasing with the increasing number of malicious clients. The graph represents the convergence performance is badly influenced if the malicious clients are present in the network. Initially, when the malicious clients are in a lower number, e.g., 2 malicious clients, the cloud server cannot identify the fake parameters, because the honest clients are much more than malicious clients. When we set the malicious clients to 4, the learning accuracy drops up to 40%. This performance drop is caused by the higher fake values that the cloud server cannot identify. In the third and fourth scenarios, we set the malicious clients to 6 and 8. In these settings, the cloud server is entirely unable to identify the system parameters and draws a constant value of accuracy, which is almost zero. The experiment proves that the higher number of malicious clients can ultimately disturb the system performance. In real-world scenarios, the activities from malicious clients might be utterly different from our settings. In this way, we cannot guarantee the actual performance drop in the real-world attacks. However, the reduction in convergence speed, i.e., the classification accuracy, i.e., the communication rounds between the clients and the cloud server, and the learning accuracy, could be badly harmed in the presence of malicious clients.

#### 5.2.2. Solution

In order to prevent data leakage from malicious clients, it is fundamental to investigate the clients and recognize the malicious ones during the initialization phase. Such clients’ recognition can be done through machine learning techniques, such as: a supervised learning technique can be implemented, which execute at the beginning of each communication round to find the malicious client in the network. A cloud server can also exploit the relationship between the weight updates at the beginning of each communication round and finds the difference in the next communication round. In this way, a malicious client will be more concerned about the performance, which results in fewer malicious activities.

### 5.3. Local Updates Phase: A Privacy and Security Threat

During the training phase, the adversaries can launch an inference attack as the clients’ exchange gradients between each other in order to compute the update, which could cause a serious privacy issue. As described in Section 3.2, local model updates can leak unintended additional information regarding the individual’s training data to the adversaries. Therefore, continuous observation on the local updates can cost a significant amount of private information such as class representatives and the properties associated with the subset of training data. After computing the local model updates, the malicious clients can launch a poisoning attack before sending the updates to the cloud server. In particular, the adversary can insert a hidden backdoor into its local update, which can poison the global model update. In the past, researchers have investigated this point that the single-sample of local updates sent to the cloud server at any communication round is poisoned, and these poisoned updates can be created through the hidden backdoor into a model [43]. The poisoning attacks on local updates can harm worse than the poisoning attacks at the initialization phase, because the adversary can launch stealth mode to avoid detection. The adversary can use an alternative minimization strategy in order to optimize the training loss and achieve the adversarial objective.

#### 5.3.1. Experiment

In the local model updates, two possible attacks could harm the convergence and privacy of clients. During the execution of FL protocol, clients kept exchanging the gradients and uploading the local models throughout the communication rounds. Therefore, the adversary has multiple chances to attack the privacy or inject a backdoor. To prove this, we deploy 20 clients, in which 5 of them are malicious, which try to launch the poisoning attacks and inference attacks.

**Figure 4:**
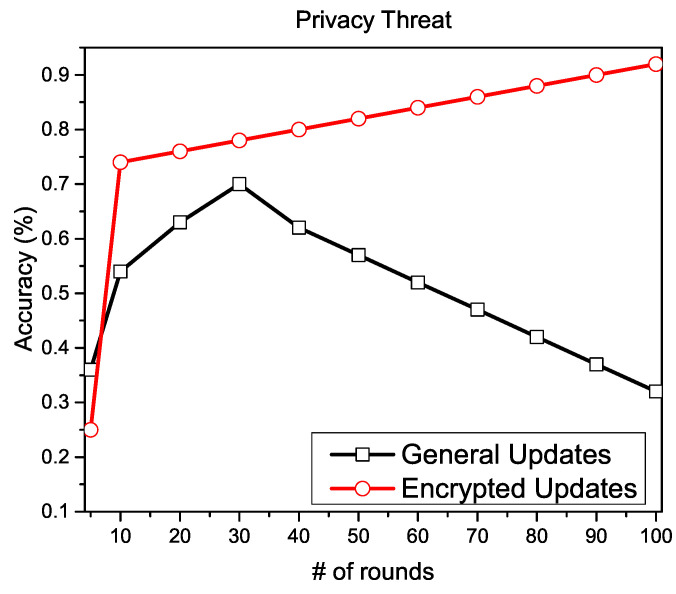
Convergence with and without cryptography.

For the privacy perspective, we apply additively homomorphic encryption on each local gradient and compare the convergence speed with general FL settings. In Figure 4, we present the convergence speed with and without the encryption of local gradients. The graph shows that the convergence is badly affected without any encryption, whereas the encrypted local gradients achieve higher convergence in the presence of malicious clients.

**Figure 5:**
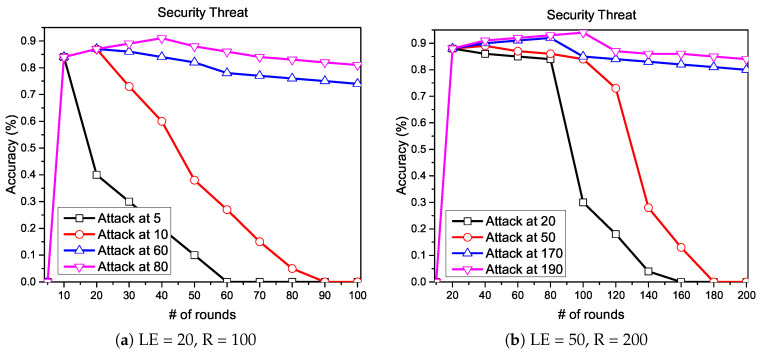
Backdoor injected at various numbers of communication rounds.

For the security perspective, we first encrypt the gradients to obtain protection against poisoning attacks and then we run the experiment for two different number of local epochs LE={20,50} and communication rounds R={100,200}, respectively. In Figure 5, we measure the convergence while injecting a backdoor at various numbers of communication rounds. In particular, in Figure 5a, we choose 100 communication rounds with 20 local epochs and then injected a backdoor at various rounds of training R={5,10,60,80}. Similarly, in Figure 5b, we choose 200 communication rounds with 50 local epochs and then injected a backdoor at various rounds of training R={20,50,170,190}. Both of the graphs in Figure 5 show that the backdoor’s impact at the early stages is higher than the backdoor at the later stages of training.

#### 5.3.2. Solution

The privacy of an individual’s data can be secured against poisoning attacks. Especially during the exchange of gradients by applying CT techniques or adding artificial noises while using DP in each local gradient, which has been adopted by plenty of existing research. These attacks still need to be investigated in detail, as the inference attacks may leak the private information of individual clients during the exchange of local updates. Additionally, injecting backdoor attacks may reduce the performance of the targeted task. However, the backdoor attack launch at early rounds tends to be forgotten with the running rounds as the new information comes with the new round. In addition, in the early training epochs, the global model focuses on learning common patterns that are shared by the clients, such as image shapes and frequent words. Even when the attack launch in the later rounds of training, at this stage, the clients share idiosyncratic features of their data, so the impact of injected backdoor can have less effect on the global weight. Therefore, it is recommended to run the FL protocol with a higher number of local epochs and global communication rounds.

### 5.4. Model Aggregation Phase: A Security Threat

After receiving the local updates from clients, the cloud server aggregates those updates in order to obtain a new global model. To achieve the desired convergence, this phase of FL is equally important as the other phases, because FL requires a new global model in each communication round. As the cloud server is the only entity to perform the aggregation process, the client’s data are protected against the internal adversaries, but still at risk from the malicious cloud server. Therefore, the clients need to apply DP or CT on the local updates before sending them to the cloud server. Extensive research has been conducted, where the authors applied additional security features in order to protect the local updates from the cloud server and achieved significant performance, as given in Table 1. However, when the clients apply additional security techniques, the cloud server cannot execute the conventional averaging process. The reason behind this phenomenon is that the cloud server requires additional computation in order to face the added noise that is generated by DP or it needs some additional time to decrypt the encrypted updates through CT. This computation cost increases linearly with the increasing number of clients. When the cloud server receives the local updates, it becomes difficult to distinguish between the perturbed update and non-perturbed update; hence, the cloud server spends an equal amount of computation resources on each update, which results in poor performance.

#### 5.4.1. Experiment

**Figure 6:**
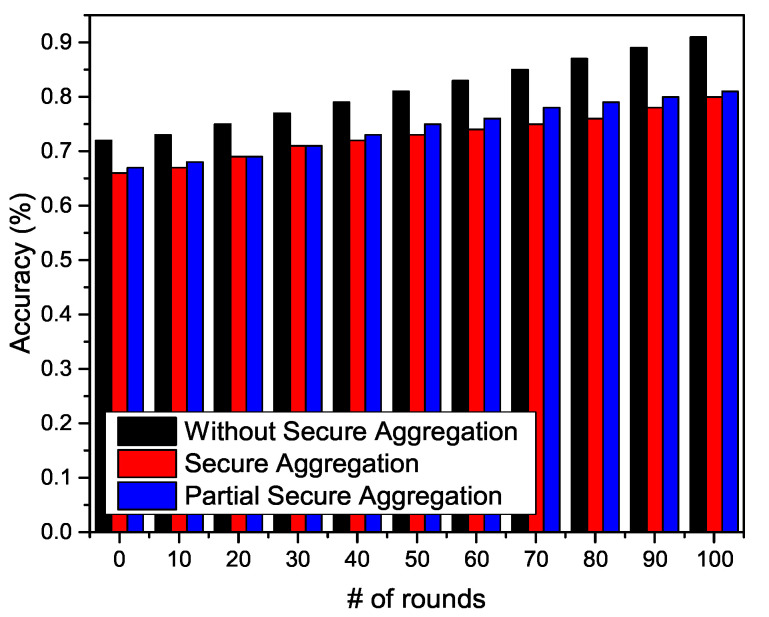
Model aggregation in three different scenarios.

We conduct the experiments for three different scenarios in order to verify the performance disturbed by the aggregator. In particular, we deploy 20 clients in traditional FL settings and run the experiment for 100 communication rounds with 20 local epochs. In the first scenario, we run the experiment without secure aggregation, and the cloud server obtains a global model, as in traditional FL settings. In the second scenario, we run the experiment with secure aggregation, where we apply DP on each local update. The cloud server aggregates the perturbed local models to obtain a global model. In the third scenario, we apply partial secure aggregation, as the DP is applied on only 10 clients, and the other 10 clients upload the updates without perturbation. In Figure 6, the performance is better without secure aggregation, as there is no additional computation cost. However, the performance with secure aggregation and partial secure aggregation is almost the same. Once the cloud server receives the perturbed update, it assumes that the next update will also be perturbed. Another reason behind this same performance is that the cloud server aggregator is not intelligent enough to distinguish between the perturbed and non-perturbed update.

#### 5.4.2. Solution

It is important to design an intelligent model aggregator in order to enhance the performance at the model aggregation phase. This intelligent model aggregator should tackle the large client scenario where the huge noises are added with the local updates. Additionally, an intelligent aggregator should distinguish between the updates and apply the aggregation method accordingly. Usually, in the traditional FL settings, the aggregation weight depends on the size of training, where the intelligent aggregator should be designed for multiple purposes. In [44], the authors proposed an intelligent aggregation method in order to address the problem of malicious clients. The authors also add a test process on the server, where the aggregator runs the test performance based on the uploaded parameters from the individual client.

### 5.5. Convergence: Affected by Privacy Threat

In the literature, several researchers provide an approximate theoretical convergence guarantee to some extent [45]. However, the existing literature considered unrealistic scenarios, such as an independent and identical distribution (IID) of data or the 100% participation of clients in each communication round. These scenarios cannot be considered at a practical level, because, in the real world, the data distribution cannot be IID. Additionally, when the perturbation method is applied to the updates, the learning parameters become non-IID. Therefore, it is also important to provide theoretical results with the privacy-protection mechanism in FL. The tradeoff between privacy and convergence should also be investigated. In order to provide a concrete example for convergence, we consider our previous work [27] that is based on IID distribution, where we apply additively homomorphic encryption at the client and DP at the cloud server. Here, we investigate the influence of aggregated noise, which results in updated non-IID system parameters. Therefore, we cannot provide a complete guarantee of convergence. The algorithm still suffers from efficient convergence in communication rounds, even when the convergence is satisfied.

#### 5.5.1. Experiment

**Figure 7:**
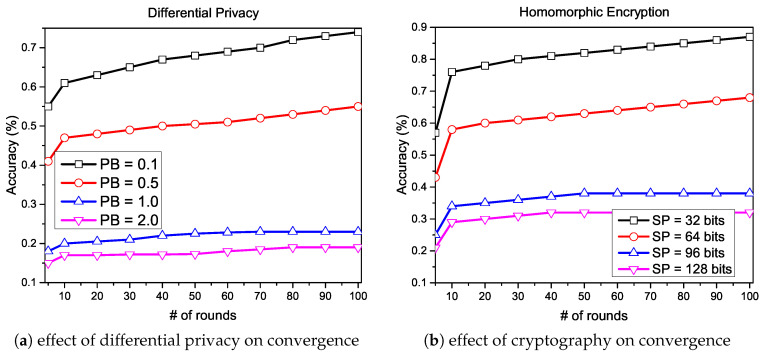
Effect on convergence with the increasing value of privacy and security.

The convergence can only be affected at the initialization phase or the local update phase, so it is essential to apply the privacy protection mechanism in these phases. Several privacy protection techniques have been adopted and proposed in the past, which demands additional operations from the cloud server, as described earlier; consequently, the computational cost is increased. We consider two different scenarios in our experiments in order to verify the convergence loss. In the first scenario, we apply DP on each local client with four different privacy budget values: PB={0.1,0.5,1,2}, and observe the difference between the increasing value of privacy on convergence. In the second scenario, we apply CT (additively homomorphic encryption) on each client with four different security parameter values: SP={32,64,96,128} in bits, and observe the difference between the increasing value of the security on convergence. We show the achieved convergence in the presence of additional noises from DP and security parameters from CT in Figure 7a,b, respectively. Both graphs presented in Figure 7 indicate that convergence speed in terms of accuracy is better when the amount of privacy and security are higher in numbers; in contrast, a lower amount of privacy and security shows poor performance.

#### 5.5.2. Solution

A higher level of privacy and security results in less level of convergence because computation cost creates a fundamental tradeoff between privacy and performance, as described above. Therefore, it is essential to investigate the optimal level of privacy budget and security parameters, which should be suitable to standard FL settings and show significant performance in large clients scenario.

## 6. Discussion Future Directions

This paper has discussed privacy and security threats in FL protocol, which should be considered while implementing a secure FL. However, besides these threats, there are various open challenges and directions that still need to be discovered properly. Below, we define the potential and promising future directions in FL.

- **Incremental Federated Learning**: training on available datasets is limited to standard machine learning techniques. However, in most real applications, the datasets are not fixed, and the clients do not participate in training due to personalized datasets. Therefore, it is important to investigate the incremental learning, where the clients can train the same model on their own dataset. Additionally, efficient convergence on such models is another open challenge that will help FL to be implemented widely.
- **Hierarchical Federated Learning**: decomposition of FL tasks into a hierarchy of subtasks, so that higher-level parent-task invokes the lower-level child-task to perform primitive actions can bring robustness in overall performance of FL. The local epochs can further decompose in sub-local epochs to make a hierarchy and perform the FL tasks hierarchically for vigorous training. The privacy and security threats in hierarchical FL will be more exciting to investigate. The computation cost should also need to be considered while implementing hierarchical FL, as the decomposition might overload the clients.
- **Mobile Federated Learning**: generally, the clients in FL training are assumed to be stable with their constant geographic location. However, in the real world, this assumption may not be applied as the latest IoT smart devices are easy to move, and the clients have kept those devices with them. Thus, training on those devices with constant geographic location is impractical. Therefore, mobile federated learning should be considered for practical implementations.
- **Decentralized Federated Learning**: in the traditional FL settings, an untrusted cloud server is required for system initialization and global model aggregation. However, it would be an interesting study to eliminate this third party, and the clients elect themselves as a cloud server in a round-robin schedule. This technique will minimize the threats from the third party. However, malicious clients may have more chances to access the individual’s private information, especially when the malicious client acted as a server in the most recent round. Therefore, the privacy-preserving technique of decentralized FL should also be investigated.
- **Adaptive Clustering**: the work-load of individual clients can be divided into multiple clients through clustering, which can bring robustness in FL’s communication efficiency. In this system, one client can become a cluster-head and be responsible for communicating with the cloud server. In contrast, the other clients in the same cluster should only communicate and forward their local updates to their cluster-head. The selection mechanism of such clients can be done based on their previous updates and their available resources. The energy consumption of individual clients can be greatly reduced by forwarding the local updates at a minimum distance. However, sharing local updates in the cluster can bring higher privacy risks, as the malicious clients can be available at any cluster and so leak the individual’s private information.
- **Clients Heterogeneity**: in the general architecture of FL, the clients are considered to be homogeneous, which hinders FL from being implemented in many real applications. In real scenarios, the clients can be different from each other in many ways, such as: federation capacity, privacy requirements, reliability, and accessibility. Therefore, it is important to consider these practical scenarios in FL. Additionally, in real-applications, the number of clients may not be fixed, and training participation could be unstable. Thus, such a system should also support dynamic scheduling, which can adjust the learning strategy in the case of participation instability.

## 7. Conclusions

In this paper, we identify potential privacy and security threats in federated learning. In particular, we show the possible threats and attacks on each of the FL execution phase. We have conducted extensive experiments on a publicly available dataset to prove the significance of those threats in order to provide concrete evidence. In addition, we provide possible solutions to protect the individual’s private information while maintaining efficiency in the network. In the end, we provide a few promising future directions that need to be investigated for the broad deployment of FL systems.

## Figures and Tables

**Table 1:** Summary of existing literature on privacy and security concerns in FL. Here, NN denotes Neural Network, LM denotes Linear Model, DT denotes Decision Tree, SA denotes Secure Aggregation, DP denotes Differential Privacy, and CT denotes Cryptographic Technique.

Existing Literature	FL Classification	Model Used	Network Architecture	Privacy Mechanism
FedCS [19]	HFL	NN	centralized	general
BlockFL [20]	HFL	LM	centralized	general
Tree-based FL [21]	HFL	DT	distributed	DP
Adversarial Lens [22]	HFL	NN	centralized	general
Secure Aggregation [23]	HFL	NN	centralized	CT
FedXGB [24]	HFL	DT	centralized	CT
SecureBoost [25]	VFL	DT	centralized	CT
q-FedAvg [26]	HFL	NN, LM	centralized	general
FedOpt [27]	HFL	NN, LM	centralized	DP, CT
Byzantine-Robust FL [28]	HFL	NN	Centralized	None
Local DPFL [29]	HFL	NN, LM	centralized	DP
Distributed Backdoor [30]	HFL	NN	centralized	general
SimFL [31]	HFL	DT	distributed	hashing
FedProx [32]	HFL	general	centralized	general
Reputation FL [33]	HFL	LM	centralized	general
Hybrid FL [34]	HFL	NN, LM	centralized	DP, CT
BREA [35]	HFL	NN	centralized	SA
FedBCD [36]	VFL	NN	centralized	general
FL-LSTM [37]	HFL	NN	centralized	DP
FedForest [38]	HFL	DT	centralized	CT
Logistic Regression FL [39]	HFL	LM	centralized	CT
Linear Regression FL [40]	VFL	LM	centralized	CT
PPRR [41]	HFL	LM	centralized	CT
Ridge Regression FL [42]	HFL	LM	centralized	CT
Backdoor FL [43]	HFL	NN	centralized	general

**Table 2:** Properties of federated learning phases with respect to privacy and security issues.

Sr #	FL Phase	Issue	Target		Attacks	Attacker	
			Model	Training Data		Participant	Server
1	Initialization	Privacy	No	Yes	Inference Attacks	Yes	Yes
2	Local Updates	Security				Yes	No
3	Model Aggregation	Security	Yes	No	Poisoning Attacks	No	Yes
4	Convergence	Privacy				Yes	No

**Table 3:** Hyper-parameters.

Parameter	Value
Network Size	$100\times 100\;m^{2}$
Global rounds	100
Local epochs	20
Learning rate	0.05
Non-IID degree	0.5
Client transmission power	200 mW
Local update size	20,000 nats
Mini-batch size	32

**Table 4:** The DNN architecture for the MNIST dataset used in the experiments (ReLu stands for Rectified Linear Unit).

Type of Layer	Layer Size
Convolution + ReLu layer 1	5×5×30
Max pooling layer 1	3×3
Convolution + ReLu layer 2	5×5×50
Max pooling layer 2	3×3
Fully connected + ReLu layer	220
